# Novel Deleterious nsSNPs within* MEFV* Gene that Could Be Used as Diagnostic Markers to Predict Hereditary Familial Mediterranean Fever: Using Bioinformatics Analysis

**DOI:** 10.1155/2019/1651587

**Published:** 2019-06-04

**Authors:** Mujahed I. Mustafa, Tebyan A. Abdelhameed, Fatima A. Abdelrhman, Soada A. Osman, Mohamed A. Hassan

**Affiliations:** ^1^Department of Biochemistry, University of Bahri, Sudan; ^2^Department of Biotechnology, Africa City of Technology, Sudan

## Abstract

**Background:**

Familial Mediterranean Fever (FMF) is the most common autoinflammatory disease (AID) affecting mainly the ethnic groups originating from Mediterranean basin. We aimed to identify the pathogenic SNPs in MEFV by computational analysis software.

**Methods:**

We carried out in silico prediction of structural effect of each SNP using different bioinformatics tools to predict substitution influence on protein structure and function.

**Result:**

23 novel mutations out of 857 nsSNPs are found to have deleterious effect on the MEFV structure and function.

**Conclusion:**

This is the first in silico analysis of MEFV gene to prioritize SNPs for further genetic mapping studies. After using multiple bioinformatics tools to compare and rely on the results predicted, we found 23 novel mutations that may cause FMF disease and it could be used as diagnostic markers for Mediterranean basin populations.

## 1. Introduction

Familial Mediterranean Fever is an autosomal recessive inherited inflammatory disease [1–3] (however, it has been observed that a substantial number of patients with clinical FMF possess only one demonstrable* MEFV* mutation [4, 5]) that is principally seen in different countries [6–10]. However, patients from different ethnicities (such as Japan) are being increasingly recognized [2, 11], and the carrier frequency for* MEFV* genetic variants in the population in the Mediterranean basin is about 8% [12]. Most cases of FMF usually present with acute abdominal pain and fever [1, 3, 7], both of which are also the main causes of referral in the emergency department [13]. All these factors may help in medical treatment. Colchicine is the first line therapy [14], but in resistant cases (<10% of patients) [15], it affects the responsiveness to Colchicine [16]; other anti-inflammatory drugs can be used for extra anti-inflammatory effect [17]. If FMF is not treated, it may be an etiologic factor for colonic LNH in children [18].* MEFV* gene is localized on 16p13.3 of chromosome 16 at position 13.3 which consists of 10 exons with 21600 bp [3, 19]. The disease is characterized by recurrent febrile episodes and inflammation in the form of sterile polyserositis. Amyloid protein involved in inflammatory amyloidosis was named AA (amyloid‐associated) protein and its circulating precursor was named SAA (serum amyloid‐associated). Amyloidosis of the AA type is the most severe complication of the disease. The gene responsible for FMF,* MEFV*, encodes a protein called pyrin or marenostrin and is expressed mainly in neutrophils [3, 19].

The definition of the* MEFV* gene has permitted genetic diagnosis of the disease. Nevertheless, as studies have unwrapped molecular data, problems have arisen with the clinical definitions of the disease [20]. FMF is caused by mutations in the* MEFV* missense SNPs (we were focusing on SNPs which are located in the coding region because it is much important in disease causing potential, which are responsible for amino acid residue substitutions resulting in functional diversity of proteins in humans) [20] coding for pyrin, which is a component of inflammasome functioning in inflammatory response and production of interleukin-1*β* (IL-1*β*). Recent studies have shown that pyrin recognizes bacterial modifications in Rho GTPases, which results in inflammasome activation and increase in IL-1*β*. Pyrin does not directly recognize Rho modification but probably is affected by Rho effector kinase, which is a downstream event in the actin cytoskeleton pathway [19, 21, 22].

The aim of this study was to identify the pathogenic SNPs in* MEFV* using in silico prediction software and to determine the structure, function, and regulation of their respective proteins. This is the first in silico analysis in* MEFV* gene to prioritize SNPs for further genetic mapping studies. The usage of in silico approach has strong impact on the identification of candidate SNPs since they are easy and less costly and can facilitate future genetic studies [23].

## 2. Method

### 2.1. Data Mining

The data on human* MEFV* gene was collected from National Center for Biological Information (NCBI) website [24]. The SNP information (protein accession number and SNP ID) of the* MEFV* gene was retrieved from the NCBI dbSNP (http://www.ncbi.nlm.nih.gov/snp/) and the protein sequence was collected from Swiss Prot databases (http://expasy.org/) [25].

### 2.2. SIFT

SIFT is a sequence homology-based tool [26] that sorts intolerant from tolerant amino acid substitutions and predicts whether an amino acid substitution in a protein will have a phenotypic effect. It considers the position at which the change occurred and the type of amino acid change. Given a protein sequence, SIFT chooses related proteins and obtains an alignment of these proteins with the query. Based on the amino acids appearing at each position in the alignment, SIFT calculates the probability that an amino acid at a position is tolerated conditional on the most frequent amino acid being tolerated. If this normalized value is less than a cutoff, the substitution is predicted to be deleterious. SIFT scores <0.05 are predicted by the algorithm to be intolerant or deleterious amino acid substitutions, whereas scores >0.05 are considered tolerant. It is available at (http://sift.bii.a-star.edu.sg/).

### 2.3. PolyPhen-2

It is a software tool [27] to predict possible impact of an amino acid substitution on both structure and function of a human protein by analysis of multiple sequence alignment and protein 3D structure; in addition, it calculates position-specific independent count scores (PSIC) for each of the two variants and then calculates the PSIC scores difference between the two variants. The higher a PSIC score difference is, the higher the functional impact a particular amino acid substitution is likely to have. Prediction outcomes could be classified as probably damaging, possibly damaging or benign according to the value of PSIC as it ranges from (0_1); values closer to zero were considered benign while values closer to 1 were considered probably damaging and also it can be indicated by a vertical black marker inside a color gradient bar, where green is benign and red is damaging. nsSNPs that is predicted to be intolerant by SIFT has been submitted to PolyPhen as protein sequence in FASTA format obtained from UniproktB/Expasy after submitting the relevant ensemble protein (ESNP) there, and then we entered position of mutation, native amino acid, and the new substituent for both structural and functional predictions. PolyPhen version 2.2.2 is available at http://genetics.bwh.harvard.edu/pph2/index.shtml.

### 2.4. Provean

Provean is a software tool [28] which predicts whether an amino acid substitution or indel has an impact on the biological function of a protein. It is useful for filtering sequence variants to identify nonsynonymous or indel variants that are predicted to be functionally important. It is available at (https://rostlab.org/services/snap2web/).

### 2.5. SNAP2

Functional effects of mutations are predicted with SNAP2 [29]. SNAP2 is a trained classifier that is based on a machine learning device called “neural network”. It distinguishes between effect and neutral variants/nonsynonymous SNPs by taking a variety of sequence and variant features into account. The most important input signal for the prediction is the evolutionary information taken from an automatically generated multiple sequence alignment. Also structural features such as predicted secondary structure and solvent accessibility are considered. If available also annotation (i.e., known functional residues, pattern, regions) of the sequence or close homologs are pulled in. In a cross-validation over 100,000 experimentally annotated variants, SNAP2 reached sustained two-state accuracy (effect/neutral) of 82% (at an AUC of 0.9). In our hands this constitutes an important and significant improvement over other methods. It is available at (https://rostlab.org/services/snap2web/).

### 2.6. PHD-SNP

An online Support Vector Machine (SVM) based classifier is optimized to predict if a given single point protein mutation can be classified as disease related or as a neutral polymorphism. It is available at (http://snps.biofold.org/phd-snp/phd-snp.html).

### 2.7. SNP&Go

SNPs&GO is an algorithm developed in the Laboratory of Biocomputing at the University of Bologna directed by Prof. Rita Casadio. SNPs&GO is an accurate method that, starting from a protein sequence, can predict whether a variation is disease related or not by exploiting the corresponding protein functional annotation. SNPs&GO collects in unique framework information derived from protein sequence, evolutionary information, and function as encoded in the Gene Ontology terms and outperforms other available predictive methods [30]. It is available at (http://snps.biofold.org/snps-and-go/snps-and-go.html).

### 2.8. P-Mut

P-MuT, a web-based tool [31] for the annotation of pathological variants on proteins, allows the fast and accurate prediction (approximately 80% success rate in humans) of the pathological character of single point amino acidic mutations based on the use of neural networks. It is available at (http://mmb.irbbarcelona.org/PMut).

### 2.9. I-Mutant 3.0

I-Mutant 3.0 is a neural network based tool [32] for the routine analysis of protein stability and alterations by taking into account the single-site mutations. The FASTA sequence of protein retrieved from UniProt is used as an input to predict the mutational effect on protein stability. It is available at (http://gpcr2.biocomp.unibo.it/cgi/predictors/I-Mutant3.0/I-Mutant3.0.cgi).

### 2.10. Modeling nsSNP Locations on Protein Structure

Project hope is a new online web-server to search protein 3D structures (if available) by collecting structural information from a series of sources, including calculations on the 3D coordinates of the protein, sequence annotations from the UniProt database, and predictions by DAS services. Protein sequences were submitted to project hope server in order to analyze the structural and conformational variations that have resulted from single amino acid substitution corresponding to single nucleotide substitution. It is available at (http://www.cmbi.ru.nl/hope).

### 2.11. GeneMANIA

We submitted genes and selected from a list of data sets that they wish to query. GeneMANIA's [33] approach is to know protein function prediction integrating multiple genomics and proteomics data sources to make inferences about the function of unknown proteins. It is available at (http://www.genemania.org/).

## 3. Results and Discussion

### 3.1. Result

**Figure 1:**
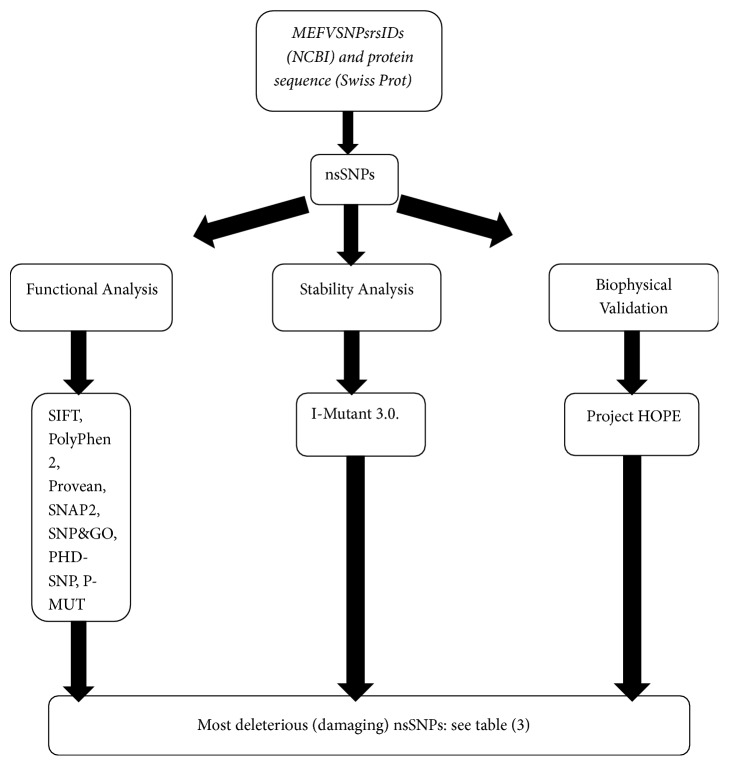
Diagrammatic representation of* MEFV* gene in silico work flow.

See Tables 1–5 and Figure 1.

## 4. Discussion

23 novel mutations have been found (see Table 3) which affected the stability and function of the* MEFV* gene using bioinformatics tools. The methods used were based on different aspects and parameters describing the pathogenicity and provided clues on the molecular level about the effect of mutations. It was not easy to predict the pathogenic effect of SNPs using single method. Therefore, multiple methods were used to compare and rely on the results predicted. In this study we used different in silico prediction algorithms: SIFT, PolyPhen-2, Provean, SNAP2, SNP&GO, PHD-SNP, P-MuT, and I-Mutant 3.0 (see Figure 1).

This study identified the total number of nsSNP in Homo sapiens located in coding region of* MEFV* gene, which were investigated in dbSNP/NCBI Database [24]. Out of 2369, there are 856 nsSNPs (missense mutations) submitted to SIFT server, PolyPhen-2 server, Provean sever, and SNAP2, respectively, and 392 SNPs were predicted to be deleterious in SIFT server. In PolyPhen-2 server, the result showed that 453 were found to be damaging (147 possibly damaging and 306 probably damaging showing deleterious). In Provean server our result showed that 244 SNPs were predicted to be deleterious. While in SNAP2 server the result showed that 566 SNPs were predicted to have effect. The differences in prediction capabilities refer to the fact that every prediction algorithm uses different sets of sequences and alignments. In Table 2 we submitted four positive results from SIFT, PolyPhen-2, Provean, and SNAP2 (see Table 1) to observe the disease causing one by SNP&GO, PHD-SNP, and P-Mut servers.

In SNP&GO, PHD-SNP and P-Mut softwares were used to predict the association of SNPs with disease. According to SNP&GO, PHD-SNP and P-Mut (70, 91 and 58 SNPs respectively) were found to be disease-related SNPs. We selected the triple disease-related SNPs only in 3 softwares for further analysis by I-Mutant 3.0, Table 3. I-Mutant result revealed that the protein stability decreased which destabilizes the amino acid interaction (S749Y, F743S, Y741C, F731V, I720T, L709R, V691G, W689R, G668R, V659F, F636C, H407Q, H407R, H404R, C398Y, H378Q, H378Y, and L86P). C375R, C395F, C395R, C395Y, and R461W were found to increase the protein stability (see Table 3).

**Figure 2:**
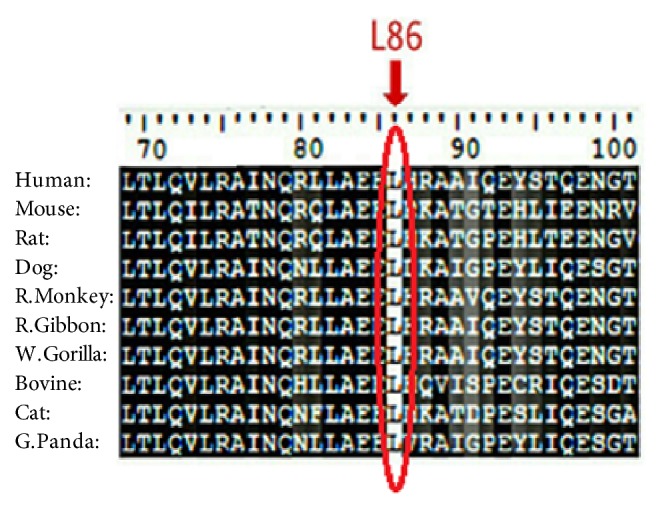
Alignments of 10 amino acid sequences of* MEFV* demonstrating that the residues predicted to be mutated in our band (indicated by red arrow) are evolutionarily conserved across species. Sequences Alignment was done by BioEdit (v7.2.5).

**Figure 3:**
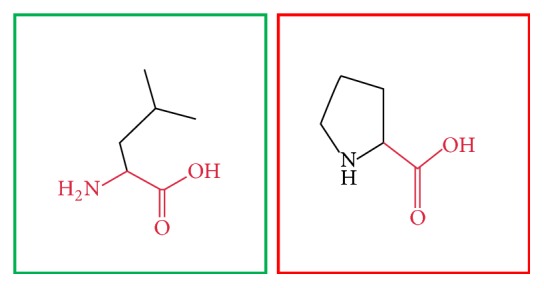
(L86P): change in the amino acid Leucine (green box) into Proline (red box) at position 86.

**Figure 4:**
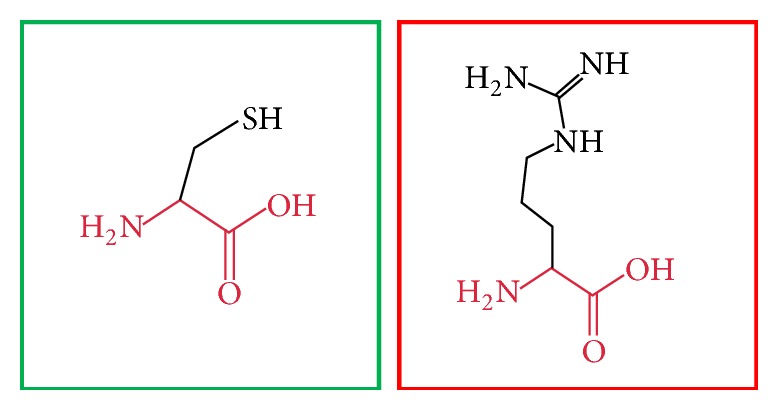
(C375R): change in the amino acid Cysteine (green box) into Arginine (red box) at position 375.

**Figure 5:**
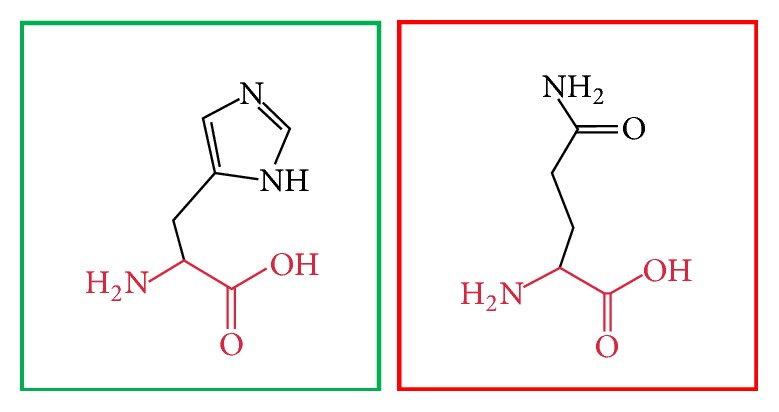
(H378Y): change in the amino acid Histidine (green box) into Tyrosine (red box) at position 378.

**Figure 6:**
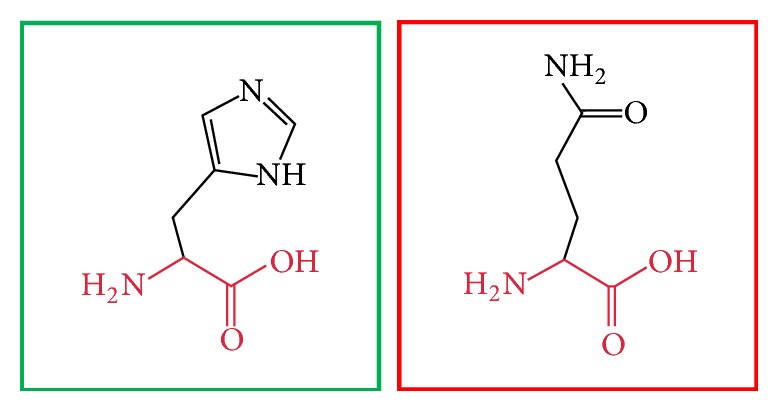
(H378Q): change in the amino acid Histidine (green box) into Glutamine (red box) at position 378.

**Figure 7:**
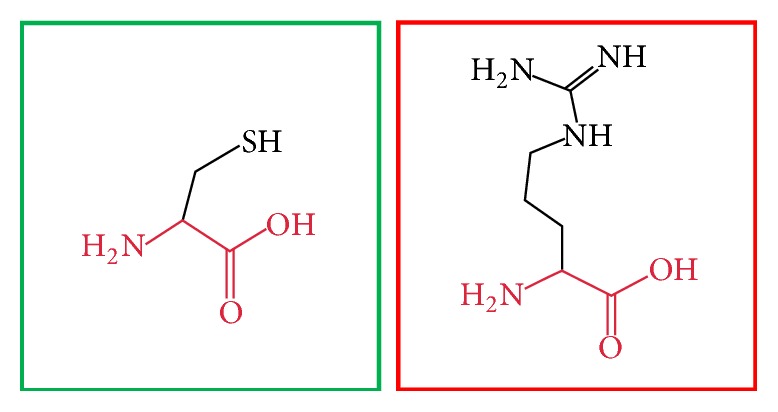
(C395R): change in the amino acid Cysteine (green box) into Arginine (red box) at position 395.

**Figure 8:**
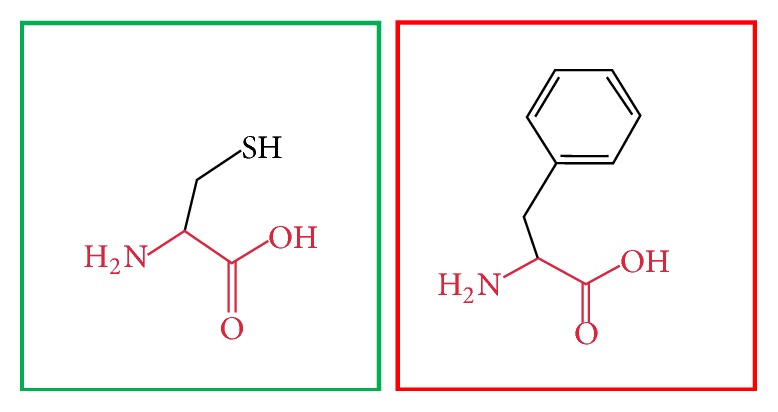
(C395F): change in the amino acid Cysteine (green box) into Phenylalanine (red box) at position 395.

**Figure 9:**
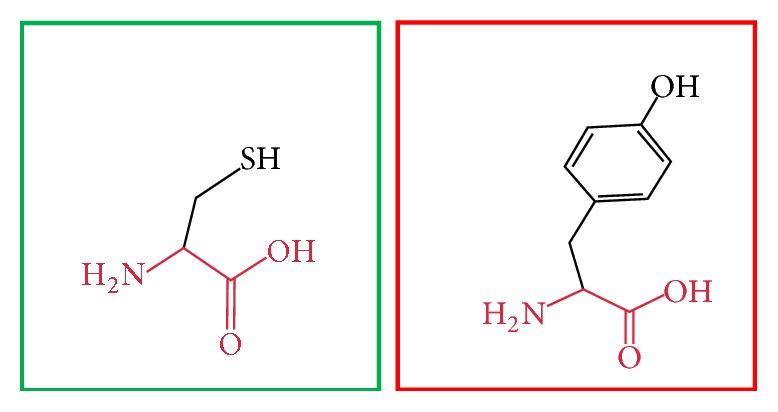
(C395Y): change in the amino acid Cysteine (green box) into Tyrosine (red box) at position 395.

**Figure 10:**
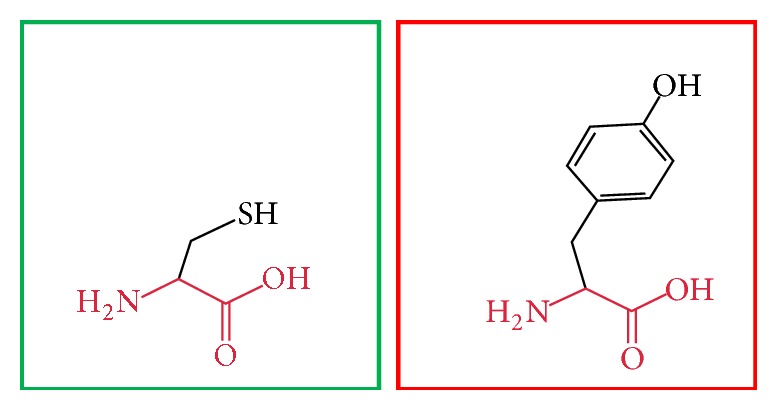
(C398Y): change in the amino acid Cysteine (green box) into Tyrosine (red box) at position 398.

**Figure 11:**
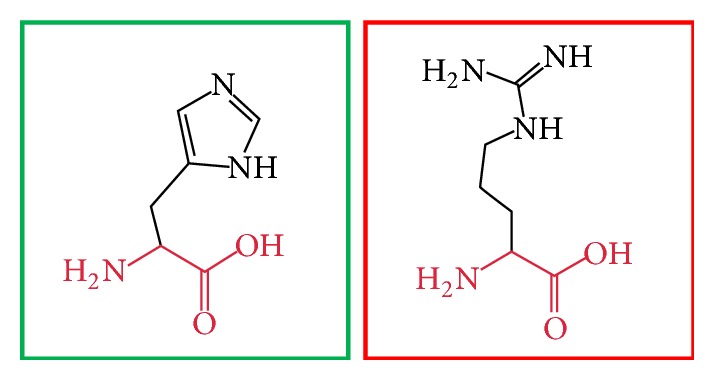
(H404R): change in the amino acid Histidine (green box) into Arginine (red box) at position 404.

**Figure 12:**
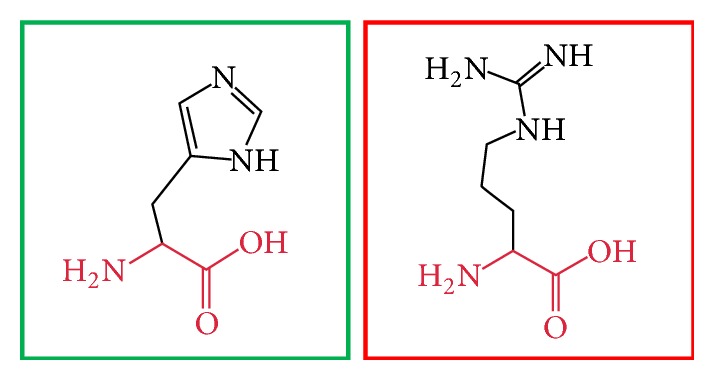
(H407R): change in the amino acid Histidine (green box) into Arginine (red box) at position 407.

**Figure 13:**
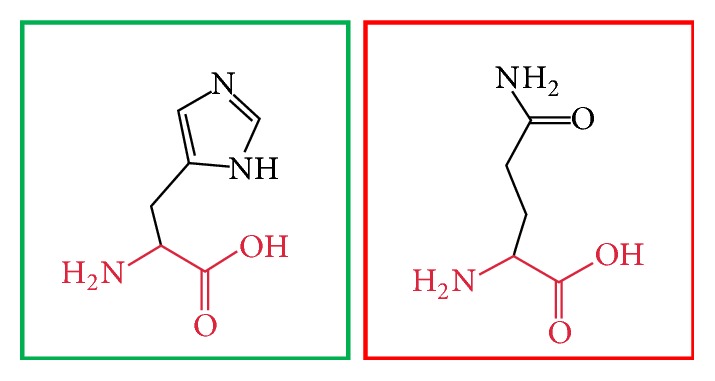
(H407Q): change in the amino acid Histidine (green box) into Glutamine (red box) at position 407.

**Figure 14:**
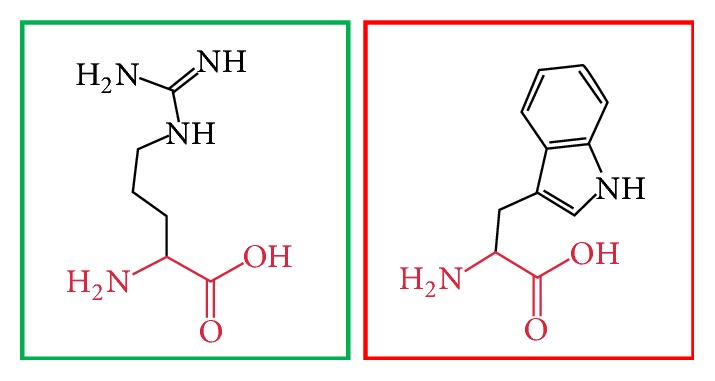
(R461W): change in the amino acid Arginine (green box) into Tryptophan (red box) at position 461.

**Figure 15:**
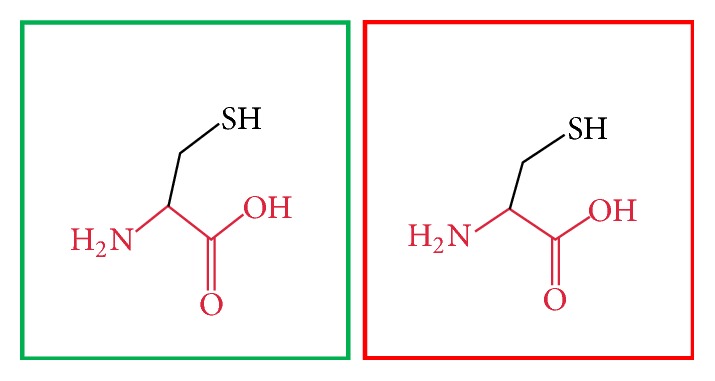
(F636C): change in the amino acid Phenylalanine (green box) into Cysteine (red box) at position 636.

**Figure 16:**
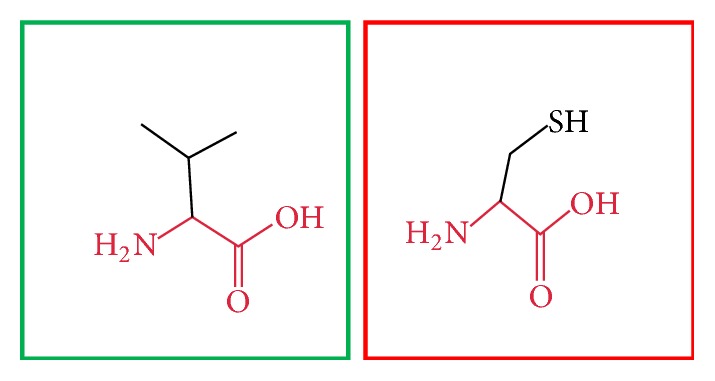
(V659F): change in the amino acid Valine (green box) into Phenylalanine (red box) at position 636.

**Figure 17:**
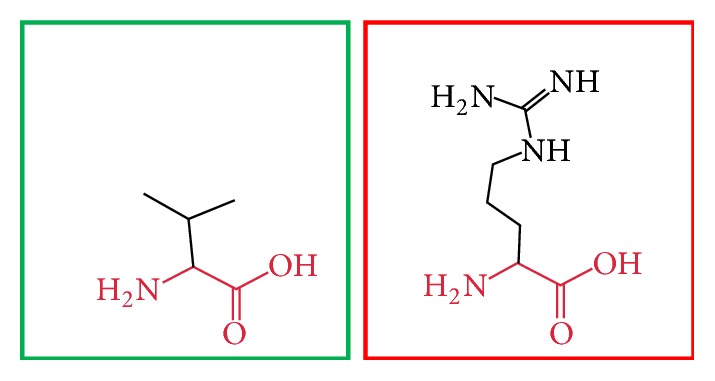
(G668R): change in the amino acid Glycine (green box) into Arginine (red box) at position 668.

**Figure 18:**
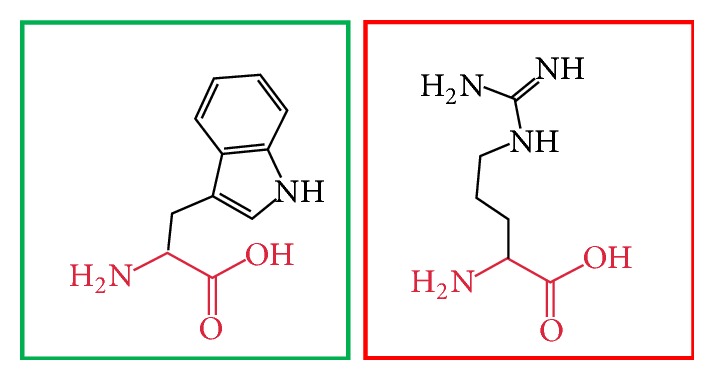
(W689R): change in the amino acid Tryptophan (green box) into Arginine (red box) at position 689.

**Figure 19:**
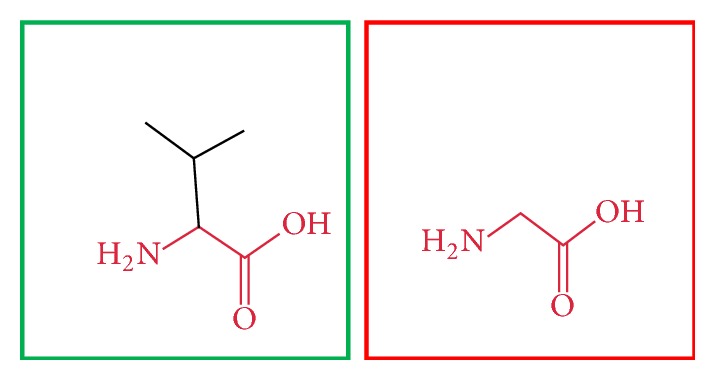
(V691G): change in the amino acid Valine (green box) into Glycine (red box) at position 691.

**Figure 20:**
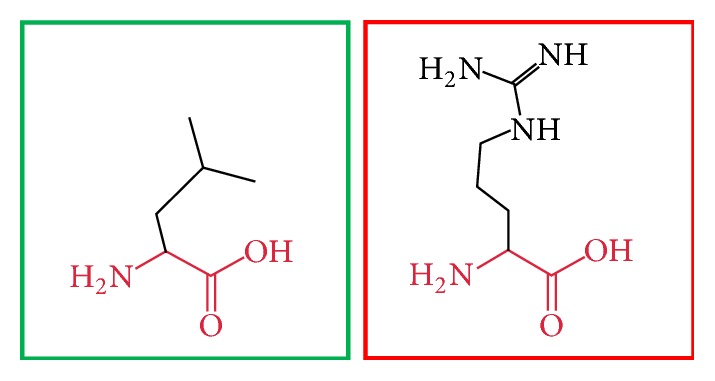
(L709R): change in the amino acid Leucine (green box) into Arginine (red box) at position 709.

**Figure 21:**
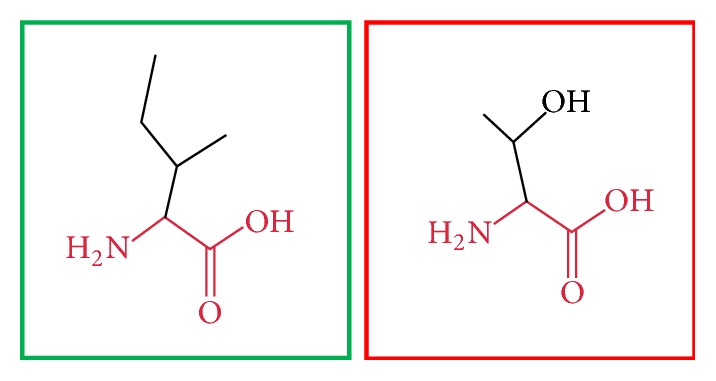
(I720T): change in the amino acid Isoleucine (green box) into Threonine (red box) at position 720.

**Figure 22:**
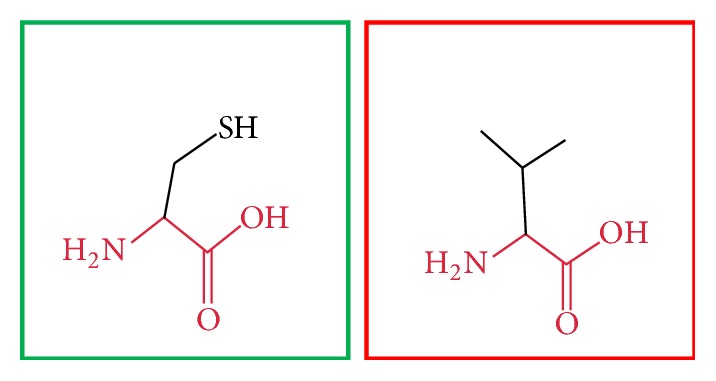
(F731V): change in the amino acid Phenylalanine (green box) into Valine (red box) at position 731.

**Figure 23:**
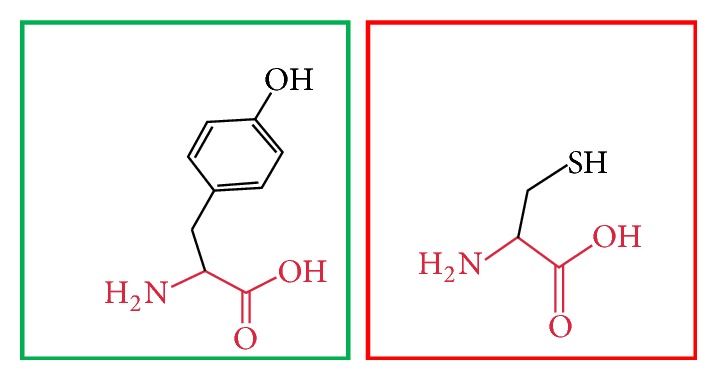
(Y741C): change in the amino acid Tyrosine (green box) into Cysteine (red box) at position 731.

**Figure 25:**
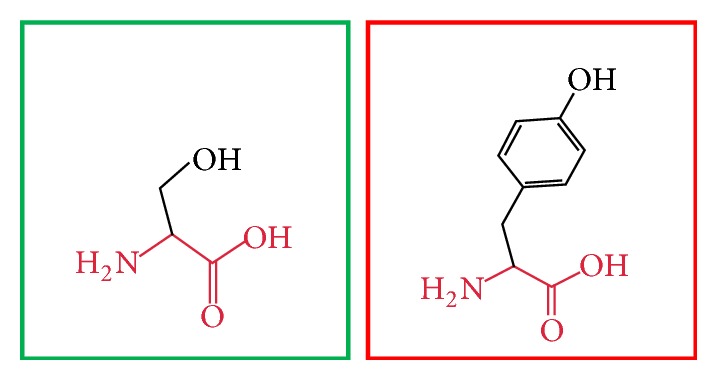
(S749Y): change in the amino acid Serine (green box) into Tyrosine (red box) at position 749.

BioEdit software was used to align 10 amino acid sequences of MEFV demonstrating that the residues predicted to be mutated in our band (indicated by red arrow) are evolutionarily conserved across species (see Figure 2). While Project HOPE software was used to submit the 23 most deleterious and damaging nsSNPs (see Figures 3–25), L86P: Proline (the mutant residue) is smaller than Leucine (the wild-type residue); this might lead to loss of interactions. The wild-type and mutant amino acids differ in size. The mutation is located within a domain, annotated in UniProt as Pyrin. The mutation introduces an amino acid with different properties, which can disturb this domain and abolish its function. The wild-type residue is located in a region annotated in UniProt to form an *α*-helix. Proline disrupts an *α*-helix when not located at one of the first 3 positions of that helix. In case of the mutation at hand, the helix will be disturbed and this can have severe effects on the structure of the protein.

**Figure 26:**
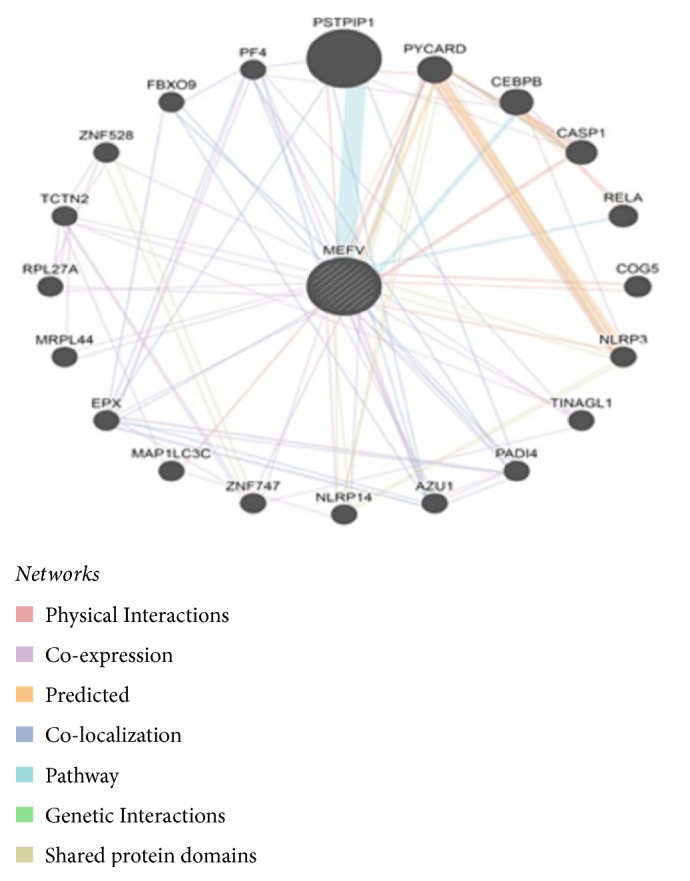
Interaction between* MEFV* and its related genes.

GeneMANIA revealed that* MEFV* has many vital functions: chemokine production, inflammatory response, interleukin-1 beta production, interleukin-1 production, intracellular receptor signaling pathway, nucleotide-binding domain, Leucine rich repeat containing receptor signaling pathway, positive regulation of cysteine-type endopeptidase activity, positive regulation of endopeptidase activity, positive regulation of peptidase activity, regulation of chemokine production, regulation of cysteine-type endopeptidase activity, regulation of endopeptidase activity, regulation of interleukin-1 beta production, regulation of interleukin-1 production, and regulation of peptidase activity. The genes coexpressed with, sharing similar protein domain, or participated to achieve similar function were shown in (see Figure 26) Tables 4 and 5.

In this study we also retrieved all these SNPs as untested (V659F, L709R, F743S, S749Y). We found it to be all damaging. Our study is the first in silico analysis of* MEFV* gene which was based on functional analysis while all previous studies [34, 35] were based on frequency. This study revealed that 23 novel pathological mutations have a potential functional impact and may thus be used as diagnostic markers for Mediterranean basin populations.

## 5. Conclusion

In this work the influence of functional SNPs in the* MEFV* gene was investigated through various computational methods, which determined that S749Y, F743S, Y741C, F731V, I720T, L709R, V691G, W689R, G668R, V659F, F636C, R461W, H407Q,, H407R, H404R, C398Y, C395Y, C395F, C395R, H378Q, H378Y, C375R, and L86P are new SNPs having a potential functional impact and can thus be used as diagnostic markers. They constitute possible candidates for further genetic epidemiological studies with a special consideration of the large heterogeneity of* MEFV* SNPs among the different populations.

## Figures and Tables

**Figure 24:**
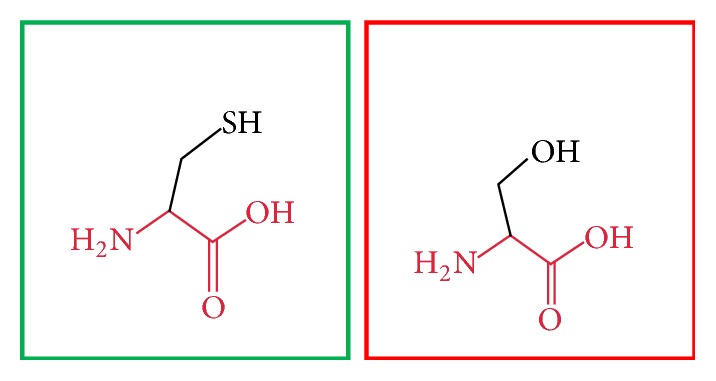
(F743S): change in the amino acid Phenylalanine (green box) into Serine (red box) at position 743.

**Table 1:** Damaging or deleterious effect nsSNPs associated variations predicted by various softwares.

*Amino Acid Change *	*SIFT*		*Polyphen*		*PROVEAN*		*SNAP2*	
	prediction	Score	Prediction	score	Score	Prediction (cutoff= -2.5)	prediction	score
S749Y	DAMAGING	0	PROBABLY DAMAGING	1.000	-3.116	Deleterious	effect	64
F743S	DAMAGING	0	PROBABLY DAMAGING	1.000	-5.563	Deleterious	effect	68
Y741C	DAMAGING	0	PROBABLY DAMAGING	1.000	-6.035	Deleterious	effect	77
F731V	DAMAGING	0	PROBABLY DAMAGING	1.000	-5.159	Deleterious	effect	81
I720T	DAMAGING	0	PROBABLY DAMAGING	1.000	-3.639	Deleterious	effect	58
L709R	DAMAGING	0	PROBABLY DAMAGING	1.000	-4.311	Deleterious	effect	77
V691G	DAMAGING	0	PROBABLY DAMAGING	1.000	-4.667	Deleterious	effect	66
W689R	DAMAGING	0	PROBABLY DAMAGING	1.000	-10.132	Deleterious	effect	89
G668R	DAMAGING	0	PROBABLY DAMAGING	1.000	-6.287	Deleterious	effect	92
V659F	DAMAGING	0	PROBABLY DAMAGING	1.000	-3.811	Deleterious	effect	64
F636C	DAMAGING	0	PROBABLY DAMAGING	1.000	-6.49	Deleterious	effect	79
R461W	DAMAGING	0	PROBABLY DAMAGING	1.000	-5.456	Deleterious	effect	68
H407Q	DAMAGING	0	PROBABLY DAMAGING	1.000	-7.335	Deleterious	effect	41
H407R	DAMAGING	0	PROBABLY DAMAGING	1.000	-7.332	Deleterious	effect	51
H404R	DAMAGING	0	PROBABLY DAMAGING	1.000	-7.349	Deleterious	effect	75
C398Y	DAMAGING	0	PROBABLY DAMAGING	1.000	-10.314	Deleterious	effect	51
C395Y	DAMAGING	0	PROBABLY DAMAGING	1.000	-10.262	Deleterious	effect	19
C395F	DAMAGING	0	PROBABLY DAMAGING	1.000	-10.315	Deleterious	effect	27
C395R	DAMAGING	0	PROBABLY DAMAGING	1.000	-11.074	Deleterious	effect	27
H378Q	DAMAGING	0	PROBABLY DAMAGING	1.000	-5.886	Deleterious	effect	38
H378Y	DAMAGING	0	PROBABLY DAMAGING	1.000	-4.884	Deleterious	effect	45
C375R	DAMAGING	0	PROBABLY DAMAGING	1.000	-8.429	Deleterious	effect	66
L86P	DAMAGING	0	PROBABLY DAMAGING	1.000	-4.1	Deleterious	effect	19

**Table 2:** Disease effect nsSNPs associated variations predicted by various softwares.

*Amino Acid Change*	SNP&GO			PHD-SNP			P-Mut	
	Prediction	RI	Probability	Prediction	RI	Score	Score	Prediction
S749Y	Disease	1	0.573	Disease	3	0.649	0.67 (85%)	Disease
F743S	Disease	2	0.617	Disease	4	0.696	0.82 (90%)	Disease
Y741C	Disease	6	0.797	Disease	7	0.869	0.61 (83%)	Disease
F731V	Disease	6	0.79	Disease	8	0.899	0.93 (94%)	Disease
I720T	Disease	6	0.811	Disease	5	0.769	0.81 (89%)	Disease
L709R	Disease	3	0.672	Disease	4	0.695	0.66 (85%)	Disease
V691G	Disease	1	0.55	Disease	3	0.675	0.92 (93%)	Disease
W689R	Disease	7	0.841	Disease	8	0.924	0.93 (94%)	Disease
G668R	Disease	6	0.778	Disease	7	0.84	0.93 (94%)	Disease
V659F	Disease	6	0.805	Disease	7	0.84	0.82 (90%)	Disease
F636C	Disease	6	0.809	Disease	7	0.86	0.60 (82%)	Disease
R461W	Disease	3	0.644	Disease	1	0.572	0.63 (84%)	Disease
H407Q	Disease	6	0.788	Disease	4	0.705	0.79 (89%)	Disease
H407R	Disease	5	0.769	Disease	3	0.673	0.86 (91%)	Disease
H404R	Disease	5	0.744	Disease	5	0.734	0.80 (89%)	Disease
C398Y	Disease	7	0.864	Disease	8	0.912	0.86 (91%)	Disease
C395Y	Disease	7	0.864	Disease	8	0.912	0.91 (93%)	Disease
C395F	Disease	7	0.859	Disease	8	0.914	0.92 (94%)	Disease
C395R	Disease	7	0.842	Disease	8	0.892	0.92 (94%)	Disease
H378Q	Disease	4	0.714	Disease	4	0.698	0.88 (92%)	Disease
H378Y	Disease	5	0.732	Disease	5	0.728	0.80 (89%)	Disease
C375R	Disease	6	0.784	Disease	6	0.822	0.92 (94%)	Disease
L86P	Disease	5	0.729	Disease	6	0.801	0.51 (79%)	Disease

**Table 3:** Stability analysis predicted by I-Mutant version 3.0 (also show the 23 novel mutations).

*Amino Acid Change*	SVM2 Prediction Effect	RI	DDG Value Prediction
S749Y	Decrease	0	-0.2
F743S	Decrease	6	-1.16
Y741C	Decrease	8	-2.5
F731V	Decrease	6	-1.52
I720T	Decrease	4	-0.92
L709R	Decrease	3	-0.56
V691G	Decrease	7	-1.25
W689R	Decrease	7	-0.73
G668R	Decrease	4	-0.37
V659F	Decrease	2	-0.19
F636C	Decrease	8	-1.28
R461W	Increase	1	-0.01
H407Q	Decrease	8	-1.48
H407R	Decrease	5	-1.1
H404R	Decrease	1	-0.06
C398Y	Decrease	2	-0.09
C395Y	Increase	4	0.26
C395F	Increase	1	0.04
C395R	Increase	4	0.13
H378Q	Decrease	4	-0.68
H378Y	Decrease	3	-0.26
C375R	Increase	2	-0.01
L86P	Decrease	2	-0.56

**Table 4:** The *MEFV* gene functions and its appearance in network and genome.

Function	FDR	Genes in network	Genes in genome
nucleotide-binding domain, leucine rich repeat containing receptor signaling pathway	1.42E-07	6	47
regulation of interleukin-1 beta production	0.000129	4	26
interleukin-1 beta production	0.000129	4	30
regulation of interleukin-1 production	0.000129	4	30
interleukin-1 production	0.000196	4	35
intracellular receptor signaling pathway	0.000201	6	207
positive regulation of cysteine-type endopeptidase activity	0.010438	4	101
positive regulation of endopeptidase activity	0.010663	4	105
positive regulation of peptidase activity	0.011	4	109
inflammatory response	0.018246	5	283
regulation of chemokine production	0.018246	3	39
chemokine production	0.022338	3	44
regulation of cysteine-type endopeptidase activity	0.033238	4	160
tumor necrosis factor production	0.033238	3	54
regulation of tumor necrosis factor production	0.033238	3	54
tumor necrosis factor superfamily cytokine production	0.0407	3	59
regulation of I-kappaB kinase/NF-kappaB signaling	0.046902	4	185
I-kappaB kinase/NF-kappaB signaling	0.057722	4	198
positive regulation of cytokine production	0.065004	4	207
positive regulation of cysteine-type endopeptidase activity involved in apoptotic process	0.099763	3	93
positive regulation of interleukin-1 beta secretion	0.099763	2	15
defense response to Gram-negative bacterium	0.099763	2	16
cysteine-type endopeptidase activator activity involved in apoptotic process	0.099763	2	17
regulation of endopeptidase activity	0.099763	4	251
glycosaminoglycan binding	0.099763	3	88
regulation of extrinsic apoptotic signaling pathway	0.099763	3	92
regulation of peptidase activity	0.099763	4	258
positive regulation of interleukin-1 secretion	0.099763	2	16
regulation of interleukin-1 beta secretion	0.099763	2	17

*∗FDR*: false discovery rate is greater than or equal to the probability that this is a false positive.

**Table 5:** The gene coexpression, shared domain, and interaction with *MEFV* gene network.

Gene 1	Gene 2	Weight	Network group
PF4	CEBPB	0.01083	Co-expression
NLRP14	MEFV	0.014663	Co-expression
EPX	PADI4	0.01094	Co-expression
CASP1	PYCARD	0.012291	Co-expression
TINAGL1	MEFV	0.021529	Co-expression
ZNF747	MEFV	0.032075	Co-expression
ZNF747	TINAGL1	0.01915	Co-expression
EPX	MEFV	0.019982	Co-expression
EPX	ZNF747	0.01848	Co-expression
MRPL44	MEFV	0.02576	Co-expression
RPL27A	MEFV	0.023047	Co-expression
TCTN2	MEFV	0.02049	Co-expression
TCTN2	ZNF747	0.021219	Co-expression
TCTN2	RPL27A	0.019574	Co-expression
ZNF528	RPL27A	0.021843	Co-expression
ZNF528	TCTN2	0.020287	Co-expression
PF4	TINAGL1	0.018596	Co-expression
PF4	EPX	0.016477	Co-expression
CASP1	PYCARD	0.005924	Co-expression
NLRP3	CEBPB	0.01342	Co-expression
CASP1	PYCARD	0.005896	Co-expression
AZU1	MEFV	0.01109	Co-expression
MAP1LC3C	NLRP14	0.011062	Co-expression
PADI4	MEFV	0.003094	Co-expression
AZU1	MEFV	0.003152	Co-expression
AZU1	PADI4	0.004853	Co-expression
ZNF747	MEFV	0.004908	Co-expression
PADI4	MEFV	0.023362	Co-expression
AZU1	MEFV	0.012616	Co-expression
AZU1	PADI4	0.014322	Co-expression
NLRP14	MEFV	0.01623	Co-expression
EPX	PADI4	0.01024	Co-expression
EPX	AZU1	0.007038	Co-expression
ZNF528	MEFV	0.039375	Co-expression
PF4	MEFV	0.017902	Co-expression
PF4	AZU1	0.012247	Co-expression
PF4	EPX	0.007715	Co-expression
TINAGL1	MEFV	0.027084	Co-expression
MRPL44	MEFV	0.011927	Co-expression
TCTN2	MEFV	0.014192	Co-expression
TCTN2	TINAGL1	0.014867	Co-expression
TCTN2	ZNF747	0.010889	Co-expression
TCTN2	MAP1LC3C	0.006994	Co-expression
TCTN2	MRPL44	0.010528	Co-expression
ZNF528	TCTN2	0.012167	Co-expression
RPL27A	MEFV	0.016846	Co-expression
TCTN2	RPL27A	0.018021	Co-expression
CASP1	PSTPIP1	0.009518	Co-expression
EPX	AZU1	0.01909	Co-localization
PADI4	MEFV	0.012301	Co-localization
PADI4	PSTPIP1	0.008748	Co-localization
AZU1	MEFV	0.011852	Co-localization
AZU1	PSTPIP1	0.008052	Co-localization
AZU1	PADI4	0.006025	Co-localization
EPX	MEFV	0.011933	Co-localization
EPX	PSTPIP1	0.008374	Co-localization
EPX	PADI4	0.006323	Co-localization
EPX	AZU1	0.006061	Co-localization
FBXO9	MEFV	0.022287	Co-localization
FBXO9	PADI4	0.009957	Co-localization
FBXO9	AZU1	0.009656	Co-localization
FBXO9	EPX	0.009948	Co-localization
PF4	MEFV	0.012063	Co-localization
PF4	PSTPIP1	0.007583	Co-localization
PF4	PADI4	0.005603	Co-localization
PF4	AZU1	0.005356	Co-localization
PF4	EPX	0.005651	Co-localization
PF4	FBXO9	0.009449	Co-localization
CEBPB	MEFV	0.159581	Pathway
RELA	MEFV	0.078321	Pathway
PSTPIP1	MEFV	0.953023	Pathway
PYCARD	MEFV	0.037199	Pathway
CASP1	PYCARD	0.037199	Pathway
CASP1	MEFV	0.469715	Physical Interactions
NLRP3	PYCARD	0.570819	Physical Interactions
PYCARD	MEFV	0.03673	Physical Interactions
PYCARD	PSTPIP1	0.028273	Physical Interactions
CASP1	PYCARD	0.017772	Physical Interactions
CASP1	CEBPB	0.010941	Physical Interactions
RELA	CEBPB	0.00247	Physical Interactions
COG5	MEFV	0.211887	Physical Interactions
NLRP3	MEFV	0.111467	Physical Interactions
MAP1LC3C	MEFV	0.104412	Physical Interactions
PYCARD	MEFV	0.292858	Physical Interactions
NLRP3	PYCARD	0.189095	Physical Interactions
PSTPIP1	MEFV	0.260595	Physical Interactions
PYCARD	MEFV	0.204673	Physical Interactions
CASP1	PYCARD	0.042335	Physical Interactions
RELA	CEBPB	0.007591	Physical Interactions
COG5	MEFV	0.387501	Physical Interactions
NLRP3	PYCARD	0.304828	Physical Interactions
NLRP3	PYCARD	1	Predicted
PYCARD	MEFV	0.455503	Predicted
CASP1	PYCARD	0.043769	Predicted
RELA	CEBPB	0.024601	Predicted
NLRP3	PYCARD	0.25852	Predicted
CASP1	CEBPB	0.445416	Predicted
CASP1	CEBPB	0.707107	Predicted
PYCARD	MEFV	0.00952	Shared protein domains
CASP1	PYCARD	0.013543	Shared protein domains
NLRP3	MEFV	0.009339	Shared protein domains
NLRP3	PYCARD	0.018527	Shared protein domains
NLRP14	MEFV	0.009512	Shared protein domains
NLRP14	PYCARD	0.018871	Shared protein domains
NLRP14	NLRP3	0.036989	Shared protein domains
ZNF528	ZNF747	0.002699	Shared protein domains
PYCARD	MEFV	0.011528	Shared protein domains
CASP1	PYCARD	0.031451	Shared protein domains
NLRP3	MEFV	0.009427	Shared protein domains
NLRP3	PYCARD	0.015448	Shared protein domains
NLRP14	MEFV	0.009815	Shared protein domains
NLRP14	PYCARD	0.016085	Shared protein domains
NLRP14	NLRP3	0.019774	Shared protein domains
ZNF528	ZNF747	0.002759	Shared protein domains

## Data Availability

The data which support our findings in this study are available from the corresponding author upon reasonable request.
